# The association between learning models and child health behaviours during the COVID-19 pandemic^☆^

**DOI:** 10.1016/j.pmedr.2025.103071

**Published:** 2025-04-14

**Authors:** Monica Prajapati, Xuedi Li, Kaylyssa Philip, Charles D.G. Keown-Stoneman, Jessica A. Omand, Alice Charach, Katherine T. Cost, Laura M. Kinlin, Leigh M. Vanderloo, Magdalena Janus, Jonathon L. Maguire, Catherine S. Birken, The TARGet Kids! Collaboration

**Affiliations:** a Dalla Lana School of Public Health, University of Toronto, Toronto, ON M5T 3M7, Canada; b Child Health Evaluative Sciences, The Hospital for Sick Children, Toronto, ON M5G 1E8, Canada; c Temerty Faculty of Medicine, University of Toronto, Toronto, ON M5S 1A1, Canada; d Li Ka Shing Knowledge Institute, St. Michael's Hospital, Toronto, ON M5B 1T8, Canada; e School of Nutrition, Toronto Metropolitan University, Toronto, ON M5B 2K3, Canada; f Department of Psychiatry, The Hospital for Sick Children, Toronto, ON M5G 1E8, Canada; g Department of Psychiatry and Behavioural Neurosciences, McMaster University, Hamilton, ON L8S 4L8, Canada; h Sickkids Research Institute, The Hospital for Sick Children, Toronto, ON M5G 0A4, Canada; i Department of Pediatrics, Faculty of Medicine, University of Toronto, Toronto, ON M5S 1A1, Canada; j School of Occupational Therapy, University of Western Ontario, London, ON N6A 3K7, Canada; k Department of Science and Evaluation, ParticipACTION, Toronto, ON M5R 1P6, Canada; l Offord Centre for Child Studies, Department of Psychiatry and Behavioural Neurosciences, McMaster University, Hamilton, ON L8S 4L8, Canada; m Department of Pediatrics, St. Michael's Hospital, Toronto, ON M5B 1W8, Canada

**Keywords:** COVID-19, Virtual learning, School learning, Physical activity, Screen time, Child health behaviours, Sleep

## Abstract

**Objective:**

This paper aimed to explore the association between school learning models (virtual vs. in-person) and child health behaviours (daily screen time, physical activity, outdoor time, sleep duration, and sleep onset time) during COVID-19, and whether these associations were modified by child's age, sex, and family income.

**Methods:**

A longitudinal cohort study was conducted among children four to 13 years from the TARGet Kids! COVID-19 Study of Children and Families between November 2020 and July 2022. TARGet Kids! is a primary care research network in Ontario, Canada. Data on sociodemographic characteristics, child school learning models and health behaviours were collected from repeated parent-reported questionnaires. Linear mixed effects models were fit adjusting for confounders identified *a priori.*

**Results:**

A total of 367 children [51 % male; 7.3 (± 2.2) years] with 779 observations on school learning model were included. Compared to in-person learning, virtual learning was associated with higher daily screen time (0.22 h; 95 % CI 0.03, 0.40), higher outdoor time (0.71 h; 95 % CI 0.56, 0.86), higher physical activity (0.64 h; 95 % CI 0.44, 0.85), and a later sleep onset time (0.22 h; 95 % CI 0.15, 0.28). Older children had higher daily outdoor time, girls had a later sleep onset time and children with a family income greater than $150,000 reported higher daily physical activity.

**Conclusions:**

Virtual learning was associated with higher daily screen time, outdoor time and physical activity, and later sleep onset time during the pandemic.

## Highlights

• COVID-19 virtual learning impact on child health behaviours in Canada is unknown.
• Virtual learning children had higher daily screen time and later daily sleep onset.
• A later sleep onset was most prominent among girls vs. boys.
• Virtual learning children had higher physical activity and outdoor time.
• Higher daily outdoor time found in children >6 years attending school virtually.

## 1 Introduction

Healthy movement behaviours enhance the physical and psychosocial health of children (Lasselin et al., 2016; Tremblay et al., 2016; World Health Organization, 2022). The World Health Organization (WHO), Canada, and other countries have adopted 24-h integrated movement behaviour guidelines for children and youth which provide age-specific recommendations for daily physical activity, sedentary behaviour, and sleep (Tremblay et al., 2016; World Health Organization, 2022). Only 4.8 % of children (five to 11 years of age) were meeting movement behaviour guidelines during the first wave of COVID-19 in Canada (Moore et al., 2020). A follow-up national study showed that during the second wave of the pandemic with only 3.1 % of this population met the guidelines (Moore et al., 2021). The pandemic impacted Canadian children through the introduction of numerous public health measures, including physical and social distancing, cancellations of organized sports, and limited playground and park use (Government of Canada, 2022). Our group showed that children's adherence to COVID-19 public health preventative measures was associated with shorter outdoor time and longer total screen time in 2020 (Li et al., 2021).

Studies have found that online learning was associated with disrupted sleep patterns, excessive screen use, and reduced physical activity in school-aged children (Katona et al., 2021; Levitt et al., 2022; Widyastari et al., 2022). All schools in Ontario were closed in March 2020, which involved the transition to virtual learning till the end of the school year in June 2020 (Gallagher-Mackay et al., 2021). In September 2020, a phased reopening occurred with optional in-person learning. From early January 2021 to September 2021, there were repeated changes from virtual learning to in-person learning across schools in Canada. Little is known about the impact of virtual learning on the health behaviours of children in Canada. Given that school children in Ontario experienced the longest school closures and transitions to virtual learning in all provinces and territories in Canada (135 school days), it is important to examine how different learning models have impacted children's health outcomes. This will inform public health guidelines and decisions regarding virtual learning during future public health emergencies and in the planning for virtual education strategies. In addition, understanding the impact of virtual learning on children's health behaviours is important for parents and educators to support and ensure the good health, growth and well-being of children.

Our study aimed to understand the impact of learning model on child health behaviours in children four to 13 years throughout the pandemic. Our primary objective was to determine whether parent-reported child learning model (virtual vs. in-person) was associated with screen time, physical activity levels, outdoor time and sleep among school-aged children in Ontario, Canada. Secondary objectives include determining whether this association differed by child age (less than six years vs. six years and older), child sex, and family income. We hypothesized that virtual learning was associated with lower physical activity, lower outdoor time, higher screen time, higher sleep duration and later sleep onset (Levitt et al., 2022; Li et al., 2021; Moore et al., 2020; Widyastari et al., 2022).

## 2 Methods

### 2.1 Study design and participants

A longitudinal cohort study was conducted in healthy children aged four to 13 years through The TARGet Kids! COVID-19 Study of Children and Families in Toronto, Canada between November 2020 and July 2022. TARGet Kids! is a practice-based research network and cohort study in Canada (www.targetkids.ca) that enrols healthy children, at ages birth to five years, from primary health care settings and follows them into adolescence (Carsley et al., 2015). Children are excluded from TARGet Kids! at enrolment if they have severe health conditions affecting growth or have severe developmental delay (Carsley et al., 2015). For the present study, children who were not yet school aged during the study (three years old or younger of age), homeschooled or were enrolled in learning pods (small groups of students learning outside the classroom but, in person) were also excluded. School-age in Canada is considered approximately four years old, when children enter kindergarten.

Participants from the TARGet Kids! cohort study were contacted to participate in our COVID-19 study in April 2020. Multiple questionnaires from the COVID-19 study measured parental report of physical and mental health, health behaviours (i.e., physical activity, outdoor play, sleep, screen time), adherence to public health measures, school and daycare attendance, and sociodemographic information. Families were invited to complete the questionnaires either over the telephone or online via REDCap (Harris et al., 2019). Informed verbal consent was obtained over the telephone from TARGet Kids! participating families.

### 2.2 Exposure variable

The primary exposure variable was parent-reported child learning model during COVID-19 (virtual or in-person). This information was obtained from a questionnaire administered at three time points: between November 2020 and May 2022 (January, June, and September; Table 1). Participants were classified into the virtual learning group if they indicated that their learning model at the time of questionnaire completion was “virtual”. Participants were classified as in-person learning if they indicated that their learning model at the time of questionnaire completion was “in-person”, or “mixed in-person and virtual.”

**Table 1:** Questions on learning model and child health behaviour measures from the TARGet Kids! COVID-19 study of children and family questionnaires used for obtaining exposure and outcome data in Canada from November 2020 to July 2022.

**Questions**	**Response Options**
***Learning Model***	
1. Which of the following best describes how your child is attending school now?	● Public School ○ If your child is currently attending Public School, is it: ■ In person ■ Virtual ■ Mixed in-person ● If mixed, what proportion of school time is in person? ____% ● Private School ○ If your child is currently attending Private School, is it: ■ In-person ■ Virtual ■ Mixed in-person and virtual ● If mixed, what proportion of school time is in person? ____% ● Homeschool (parent(s)/guardian(s) responsible for academic program) ● Private small groups or pods ● Not in school ○ Why is your child currently not attending school? ■ Kept at home ■ Dropped out ■ Unable to attend ■ School is closed ■ Child is not school aged ● Other – Please describe: ______________________________ ○ If your child is currently attending school in other format, is it: ■ In-person ■ Virtual ■ Mixed in-person and virtual ● If mixed, what proportion of school time is in person?____%
***Child Health Behaviours***	
2. During the past two weeks, on a typical day how much time did your child spend:	
2a. Doing light physical activity (e.g., walking active floor-based play, tummy time)?	Hours: ____ and Minutes: _____
2b. Doing moderate-to-vigorous physical activity (e.g., crawling, running, climbing stairs, bike riding, swimming)?	Hours: ____ and Minutes: _____
3. During the past two weeks, on a typical day, how much time did your child spend outdoors?	Hours: ____ and Minutes: _____
4. During the past two weeks, on a typical day:	
4a. What time does your child usually fall asleep for the night?	_____:_____ AM/PM
4b. What time does your child usually wake up for the day?	_____:_____ AM/PM
5. During the past two weeks, on a typical day how much time did your child spend:	
5a. Watching TV or digital media (e.g., Netflix)?	Hours: ____ and Minutes: _____
5b. Using social media (e.g., Instagram, Snapchat, Twitter, YouTube, TikTok)?	Hours: ____ and Minutes: _____
5c. Playing video games?	Hours: ____ and Minutes: _____
5d. Videochatting (e.g., Skype, FaceTime, Zoom)?	Hours: ____ and Minutes: _____
5e. *E*-learning or online school work (e.g., direct instruction via Zoom or Google Meet)?	Hours: ____ and Minutes: _____

### 2.3 Outcome variables

The primary outcome was parent-reported child daily screen time during COVID-19 and the secondary outcomes included parent-reported child physical activity time, outdoor time, sleep duration, and sleep onset during COVID-19. Information related to these outcomes was obtained from questionnaires administered bi-weekly during COVID-19. Total recreational screen time was the sum of minutes of time watching television, using social media, playing video/computer games, and video chatting (the sum of questions 5a through 5d in Table 1). Notably, time allocated for e-learning or online schoolwork was not incorporated into this definition of total recreational daily screen time as online school learning was considered virtual learning for the main exposure. Physical activity time was the sum of time spent in light, moderate, and vigorous activity daily (question 2a through 2b in Table 1). Outdoor time was the number of hours children spent outdoors in a day (question 3 in Table 1). Sleep duration was the difference between reported bed (question 4a in Table 1) and wake (question 4b in Table 1) times. Sleep onset was the time children went to bed (question 4a in Table 1).

### 2.4 Covariates

Confounders adjusted in the analysis were identified *a priori* from the literature and included child age, child sex, parent-reported family income, child's ethnicity, unemployment due to COVID-19, maternal education, and calendar date (Anderson et al., 2008; Armstrong et al., 2018; Boxberger and Reimers, 2019; Jay et al., 2020; Jones, 2017; Love et al., 2019; Moore et al., 2020; Statistics Canada, 2019; Tremblay et al., 2016; Webb Hooper et al., 2020). Parent-reported family income represents annual household income before tax in Canadian dollars. The calendar date was the date the outcome was reported and was also used as a proxy of other time-varying external changes such as public health preventative measures.

Predictors of outcome were included in the model as covariates based on the literature. Living arrangement was included as a predictor for the physical activity and outdoor time outcomes (Tremblay et al., 2016). Housing was included as a predictor for the physical activity and outdoor time outcomes and included apartment or house to account for accessibility of outdoor space (Lambert et al., 2019; Statistics Canada, 2020; Webb Hooper et al., 2020). Whether or not the child had siblings was also included as a predictor for the physical activity and outdoor time outcomes (Kracht and Sisson, 2018). Number of screen devices was included as a predictor for the screen time outcome (Arundell et al., 2020).

Child age, child sex and self-reported family income were identified *a priori* as potential effect modifiers. Child age categories identified *a priori* were children in kindergarten (under six years) and those in elementary school (six years and older). Annual parent/caregiver-reported family income in Canadian dollars was categorized into less than $80,000 (USD $56,225.18), $80,000 to $149,999 (USD $56,225.18 to $105,421.52) and $150,000 (USD $ 105,422.22) or greater.

Data for all covariates except unemployment due to COVID-19 were collected from the most recent parent-completed, standardized TARGet Kids! questionnaire adapted from the Canadian Community Health Survey before the COVID-19 pandemic (Government of Canada, 2022). Data related to unemployment due to COVID-19 were collected from questionnaires administered during the pandemic. Unemployment data due to COVID-19 was collected at the same time and through the same questionnaire as the outcome assessment.

### 2.5 Statistical analysis

Outcome data were restricted to within 90 days after the child learning-model exposure was collected to establish temporality and outliers were removed. Daily outdoor time with observations more than nine hours, screen time with observations more than 11.5 h, sleep duration with observations more than 14 h, and physical activity with observations more than 12 h were identified as outliers and removed from the analysis (Li et al., 2021). For the main analysis, unadjusted and adjusted linear mixed models were fitted for the continuous health behaviour outcomes accounting for repeated measures at the family level through random intercepts. Fixed effects estimate the overall association between the exposure and outcome, while random effects capture repeated measures within each child and family-level clustering.

Global interaction tests were used to assess the evidence for effect modification between the exposures and outcomes through child age, sex and family income (threshold of *p* < 0.3 was used) (Harrell, 2001). As self-reported family income was found to be a significant interaction (*p* = 0.016) for the daily physical activity time outcome, the adjusted model included self-reported family income as an interaction term. As child's age was found to be significant for the daily outdoor time outcome (*p* = 0.004), the adjusted model included child's age as an interaction term. As child's sex was found to be significant for the sleep onset outcome (*p* = 0.008), the adjusted model included child's sex as an interaction term.

Multiple imputations (*m =* 15) were performed with the *mice* package in R to reduce bias from missing data on sociodemographic variables (Buuren & Groothuis-Oudshoorn, 2011). Missing data were present in some covariates, including child's ethnicity (15 %), parent-reported family income (5 %), employment status (6 %), maternal education (4 %), child's living arrangement (4 %), housing status (10 %), siblings (4 %) and number of screen devices (4 %). It was assumed that data were missing at random conditional on the other variables included in the model. Primary analysis results were presented as effect estimate and 95 % confidence intervals for each outcome (Table 4). Statistical analyses were performed using R version 4.2.1 statistical software (R Core Team, 2024).

### 2.6 Ethics approval

This study was approved by the Research Ethics Boards at The Hospital for Sick Children (REB #10000–12,436) and Unity Health Toronto (REB #17–335). All procedures performed in studies involving human participants were in accordance with the ethical standards of the institutional and/or national research committee and with the 1964 Helsinki declaration and its later amendments or comparable ethical standards. Privacy rights of participants have been observed and informed consent was obtained for all participants.

## 3 Results

**Fig. 1:**
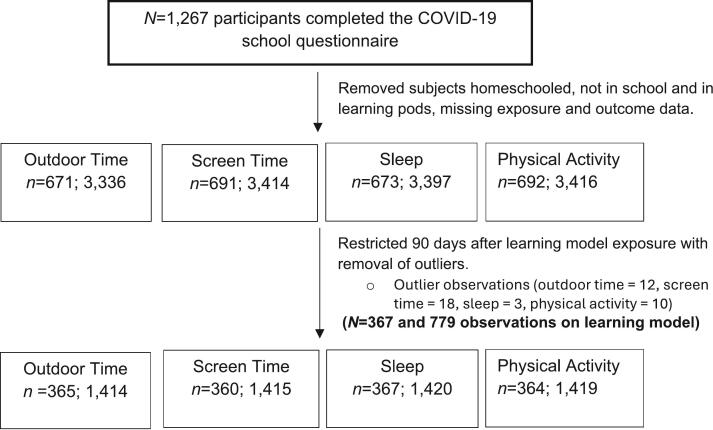
Sample size flow chart of Canadian children participants with number of observations for each child health behaviour outcome from November 2020 to July 2022.

A total of 367 children with 779 observations on learning model exposure were included in this study (Fig. 1) between November 27, 2020 to July 3, 2022. Baseline demographic characteristics of participants are presented in Table 2. The average age of children was 7.3 years (± 2.2), where 51.0 % were male and 59.0 % were of European ethnicity. More than half (58.9 %) of participants belonged to a household with a self-reported family income greater than $150,000. Of the 779 observations, 538 (69 %) and 241 (31 %) were children attending in-person and virtual school, respectively. During the COVID-19 pandemic, participants had an average daily screen time of 2.9 h (± 2.4), 3.1 h of daily physical activity time (± 2.2), 1.9 h of daily outdoor time (± 1.4), 10.3 h of sleep each day (± 0.8), mean sleep onset time at 9:00 pm (± 0.88 h) and mean wake-up time at 7:17 am (± 0.70 h) (Table 3). The assessment for multicollinearity demonstrated that collinearity was not considered to be a problem.

**Table 2:** Baseline demographic characteristics of children in Canada enrolled from the TARGet Kids! COVID-19 study of children and families from November 2020 to July 2022 (*N* = 367).

**Variables**	**All** **Mean (*****SD*****)** **or*****N*****(%)**	**%** **Missing**	**Virtual** **Mean (*****SD*****) or*****N*****(%)**	**In-Person** **Mean (*****SD*****)** **or*****N*****(%)**
**Child's Age (months)** Age range (months)	88.1 (25.9) 47–150	0	94.3 (25.6) 49–143	90.7 (25.8) 47–150
				
**Child's Sex (male)**	180 (51.0)	0	34 (45.9)	146 (52.3)
**Child's Ethnicity** African Arab East Asian European Latin American Mixed Ethnicity South Asian Southeast Asian	<5 <5 7 (2.2) 184 (59.0) 9 (2.9) 94 (30.1) 8 (2.6) 5 (1.6)	15	<5 0 0 41 (64.0) 0 19 (29.7) <5 <5	<5 <5 7 (2.8) 143 (57.7) 9 (3.6) 75 (30.2) 6 (2.4) <5
				
**Annual Self-Reported Family Income (CAD)** $0 to $79,999 $80,000 to $149,999 $150,000 or more	42 (12.0) 102 (29.1) 206 (58.9)	5	11 (15.1) 26 (35.6) 36 (49.3)	31 (11.2) 76 (27.4) 170 (61.4)
				
**Employment Status (unemployed)**	35 (10.2)	6	9 (12.2)	26 (9.7)
**Maternal Education** Apprenticeship or trades certificate or diploma College, CEGEP or other non-university certificate or diploma High school certificate or equivalent University certificate, diploma or degree	<5 29 (8.2) 12 (3.4) 307 (87.2)	4	0 8 (10.8) 5 (6.8) 61 (82.4)	<5 21 (7.6) 7 (2.5) 246 (88.5)
**Living Arrangement (2 parents in same household)**	330 (94.0)	4	69 (93.2)	261 (94.2)
**Housing (live in a house)**	293 (88.3)	10	62 (89.9)	231 (87.8)
**Siblings (yes)**	264 (74.8)	4	56 (75.7)	208 (74.6)
**Number of Screen Devices at Home**	8.4 (3.1)	4	8.6 (3.2)	8.7 (3.2)

Annual self-reported family income categories in United States Dollar are less than $56,225.18, $56,225.18 to $105,421.52 and $105,422.22 or more.

Abbreviations: CAD: Canadian dollars; CEGEP: Collège d'enseignement general et professionnel (general and professional teaching college in Quebec, Ontario).

**Table 3:** Summary of child health behaviour outcomes across learning model type among Canadian children from November 2020 to July 2022 (*N* = 367).

**Outcome**	**All** **Mean (*****SD*****)**	**All Observations**	**Virtual** **Mean (*****SD*****)**	**Virtual Observations**	**In-Person** **Mean (*****SD*****)**	**In-Person Observations**
Screen time per day (hours)	2.9 (2.4)	1415	3.0 (2.6)	429	2.8 (2.4)	986
Physical activity time per day (hours)	3.1 (2.2)	1419	3.5 (2.4)	429	2.9 (1.9)	990
Outdoor time per day (hours)	1.9 (1.4)	1414	2.4 (1.9)	426	1.7 (1.2)	988
Duration of sleep per day (hours)	10.3 (0.8)	1420	10.3 (0.8)	426	10.3 (0.8)	994
Time of sleep onset (bedtime)	21:00 (0.88)	1420	21:11 (0.92)	426	20:56 (0.86)	994
Wake up time	7:17 (0.70)	1420	7:28 (0.81)	426	7:12 (0.63)	994

From the primary adjusted analysis, (Table 4, Model 2), children who attended school virtually had higher daily recreational screen time by 0.22 h (95 % CI: 0.03, 0.40), higher daily outdoor time by 0.71 h (95 % CI: 0.56, 0.86), higher daily physical activity time by 0.64 h (95 % CI: 0.44, 0.85) and later daily sleep onset by 0.22 h (95 % CI: 0.15, 0.28) during the COVID-19 pandemic compared to those who attended school in person.

**Table 4:** Primary analysis linear mixed effect model regression results between learning model and each child health behaviour outcome in Canada from November 2020 to July 2022.

		**Model 1** **(Unadjusted)**	**Model 2**^a^ **(Adjusted)**
**Exposure Variable**	**Outcome** **Variables**	**Beta (95 % CI)**	**Beta (95 % CI)**
Virtual compared to in-person	Screen Time	0.27 (0.08, 0.45)⁎	0.22 (0.03, 0.40)⁎
		
	Outdoor Time	0.86 (0.71, 1.01)⁎	0.71 (0.56, 0.86)⁎
		
	Physical Activity	0.73 (0.53, 0.93)⁎	0.64 (0.44, 0.85)⁎
		
	Sleep Duration	−0.05 (−0.12, 0.03)	−0.01 (−0.09, 0.06)
		
	Sleep Onset	0.27 (0.19, 0.33)⁎	0.22 (0.15, 0.28)⁎

Abbreviations: CI: confidence interval.

a Adjusted for Child's age, Child's sex, Child's Ethnicity, Self-reported family income, Employment status, Maternal education, Living arrangement, Housing, Siblings, Number of screen devices at home, Outcome date.

⁎ Statistically significant.

Specifically, among children attending school virtually, those belonging to a family of self-reported family income of less than $80,000 had lower daily physical activity (β= −0.13, 95 % CI: −0.87, 0.62) compared to children of families with an income of $80,000 to $149,999 (β= 0.42, 95 % CI: 0.05, 0.78) and children of families with an income of $150,000 or more (β= 0.85, 95 % CI: 0.59, 1.11).

There was evidence suggesting that child's age modified the association between daily outdoor time and learning model. Specifically, younger children (aged five years) attending school virtually had lower daily outdoor time (β= 0.43, 95 % CI: 0.17, 0.89) compared to older children (aged 10 years old) attending school virtually (β= 0.91, 95 % CI: 0.69, 1.12).

Specifically, female children attending school virtually had later daily sleep onset time (β= 0.29, 95 % CI: 0.20, 0.38) compared to male children attending school virtually (β= 0.14, 95 % CI: 0.04, 0.23).

## 4 Discussion

In this longitudinal cohort study of children aged four to 13 years, children who attended school virtually had higher daily screen time, higher physical activity time, higher outdoor time and later daily sleep onset during the COVID-19 pandemic between November 2020 to July 2022 compared to those who attended school in-person. Among children attending school virtually, younger children had lower daily outdoor time compared to older children; children from lower income families had lower daily physical activity compared to those with a family income of $80,000 or more; girls had a later sleep onset time compared to boys.

Similar to previous research, our findings highlight that a major implication of virtual learning on child health behaviour during the COVID-19 pandemic was higher daily recreational screen time and a later sleep onset time. A cross-sectional study of children aged five to 17 years in Thailand which examined the impact of learning modes on child health behaviour also found that online learning encouraged higher screen time and disrupted sleeping patterns (Widyastari et al., 2022). A 2021 cross-sectional study from the United States found a later sleep midpoint among children aged five to 10 years receiving remote instruction during COVID-19 compared to children receiving in-person learning (Levitt et al., 2022). These findings suggest that schools may consider strategies to avoid excessive screen use among virtual learners, such as including structured breaks, engaging offline activities, and guiding parents on setting screen limits. Our results also highlight the opportunity for parents to create healthy bedtime habits and schedules for their children, encouraging children to avoid screen time before bedtime to and improve their quality of sleep.

Higher daily physical activity and outdoor time suggest that virtual learning may not have reduced the movement of children during the pandemic as much as hypothesized. A longitudinal cohort study evaluating the impact of school learning models (virtual vs. in-person school attendance throughout the pandemic) and children adherence to 24-h movement guidelines in the United States found that children aged four to 10 years attending school in-person were more likely to meet physical activity behaviour guidelines (Pfledderer et al., 2022). In contrast to our study where parent report was used to collect data, Pfledderer et al. assessed participant physical activity via wrist-worn accelerometers for 14 days (Pfledderer et al., 2022). Virtual learning may still have different implications for directly measured physical activity in children as in-person school learning environments may cause children to be more active on a short-term basis. Parents of children attending school virtually may more accurately report physical activity and outdoor time due to closer observation of child's movement behaviour compared to when children attend in-person learning environments. As parents observe higher screen time use for virtual school, they may encourage their children to become active and spend time outside of home. While there may be potential advantages of flexible learning environments, it is important for teachers to encourage outdoor breaks and incorporate physical activity into virtual lessons.

In our study, children from higher income families were found to have higher daily physical activity compared to those from lower income families. A study which investigated the association between familial factors such as family income and physical activity among children six to 17 years across the United States in 2018 showed that high family income was significantly associated with higher odds of children meeting the guideline of 60 min of daily physical activity compared to lower income groups (Burns et al., 2020). The associations differed by income levels and in the context of school closures during the pandemic, these findings raise concerns about the differential impacts of virtual learning for children from lower income families. Children from higher income families may have more access to playgrounds, fields for sports and physical activity opportunities compared to those from lower income families. Equitable access for physical activity is important for public health officials and leaders to invest in safe outdoor spaces and physical activity resources for all children.

Our study found higher daily outdoor time among older children (more than six years of age) attending school virtually. Older children may be more independent to play outdoors than younger children, who may require parental supervision. Parents may create more indoor activity opportunities for younger children to supervise them better if they are working from home or consider structured outdoor times during working hours. A 2010 Australian study of children aged five to 12 years old found that younger boys had lower outdoor time due to a higher tendency to be indoors but more social opportunities such as having friends living nearby allowed for higher outdoor time (Cleland et al., 2010). Among older boys, there were more outdoor activities available but, lack of adult supervision restricted their outdoor play time (Cleland et al., 2010). These findings align with our results and suggest that parent supervision and social opportunities are key predictors of outdoor time for different age groups. A 2023 study investigating the trends in child health behaviours among children aged nine to 12 years before, during and post the COVID-19 pandemic years in the Netherlands found that younger children spent more time playing outdoors more days every week than older children (de Bruijn et al., 2023). The mixed results on differences in outdoor time among age groups indicate a need for more nuanced research methods to establish reliable evidence to guide parents in ensuring adequate outdoor play time for their children.

Our study found a later sleep onset time in girls compared to boys in the virtual learning group. A cohort study looking at sleep-related difficulties in children aged four to nine years and adolescents aged 10–17 years in Germany found that sleep-related difficulties such as bedtime resistance and sleep onset delay were more frequent among younger boys in the child sample and among girls than boys in the adolescent sample (Lewien et al., 2021). This evidence suggests that further research stratifying child sleep by sex is important and needed to inform public health guidelines and guide parents in supporting the sleep schedules of their children. Sleep-related findings in the context of virtual learning suggest that parents should encourage an early bedtime and healthy sleep habits and schedules to avoid sleep-related difficulties.

Overall, to create a supportive learning environment at home, parents may need resources to help monitor their child's health behaviours and foster healthy habits. Teachers are advised to include wellness activities such as physical activities, frequent movement breaks and peer interactions into virtual learning environments. Collaboration between parents, educators and policymakers may be useful to help develop proactive strategies to support the health, wellbeing and education of children in different learning environments.

This study is one of the first to explore the association of school learning models and child health behaviours among children in Canada during the COVID-19 pandemic. Important strengths of this research are the use of a longitudinal cohort study design, repeated measures exploring the impact of school learning models and a diverse range of parent-reported child health behaviours during COVID-19. Our study also outlines differences by age, sex and family income levels. A major strength is that it provides evidence that can inform support of health behaviours and physical activity regardless of the mode of learning. Limitations include a small sample size and majority of participants come from high income households and maternal educational status in the Greater Toronto Area, which limits generalizability to other contexts. Also, the outcome variables were parent-reported, which may have resulted in under- and over-estimations of certain health behaviours (Rosenman et al., 2011). As there is a two-week recall period when obtaining outcome data information by parents, there may be potential recall bias affecting the internal validity of the data. Our data was limited in capturing transitions between learning models. Since there may be changes from virtual to in-person learning instructions from schools, there may be children who switched between learning models across the three time points. The accuracy of the reporting may also differ based on learning model of the child. There may be unmeasured confounding not accounted for in the analysis, and results are limited by observational nature, preventing us from making causal inferences.

## 5 Conclusion

The transition from in-person to virtual learning during the COVID-19 pandemic impacted recreational screen time, physical activity, outdoor time and sleep onset time of children. Our study's findings may provide evidence to decision makers including policymakers, teachers, and parents to consider fostering in-person learning environments that promote physical activity and outdoor time, and appropriate sleep hygiene. In the case of future public health emergencies, findings from this study may help to prepare transitions to virtual learning through the consideration and implementation of opportunities to reduce sedentary behaviour among young children, and to support equitable access to opportunities for physical activity and outdoor time for all students in virtual and in-person learning environments. Future studies are needed to assess the long-term impacts of virtual learning on child health behaviours after the COVID-19 pandemic in children.

## Author contributions

MP and XL were involved in conceptualization, investigation, methodology, formal analysis, interpretation of the data and contributed to the original draft of the manuscript and reviewed and revised the manuscript.

KP and CKS were involved in conceptualization, methodology, formal analysis, interpretation of the data and contributed to critical reviewing and editing of the manuscript.

CB and JM were involved in conceptualization, methodology, investigation, supervision, resources, funding acquisition, project administration and contributed to critical review and editing of the manuscript.

JO, AC, KC, LK, LV and MJ were involved in conceptualization, funding acquisition and contributed to critical review and editing of the manuscript.

All authors approved the final manuscript as submitted and agree to be accountable for all aspects of this work.

## CRediT authorship contribution statement

**Monica Prajapati:** Writing – review & editing, Writing – original draft, Methodology, Investigation, Formal analysis, Conceptualization. **Xuedi Li:** Writing – review & editing, Writing – original draft, Supervision, Methodology, Investigation, Funding acquisition, Conceptualization. **Kaylyssa Philip:** Writing – review & editing, Methodology, Investigation, Conceptualization. **Charles D.G. Keown-Stoneman:** Writing – review & editing, Methodology, Funding acquisition, Data curation, Conceptualization. **Jessica A. Omand:** Writing – review & editing, Funding acquisition, Conceptualization. **Alice Charach:** Writing – review & editing, Funding acquisition, Conceptualization. **Katherine T. Cost:** Writing – review & editing, Investigation, Funding acquisition, Conceptualization. **Laura M. Kinlin:** Writing – review & editing, Investigation, Funding acquisition, Conceptualization. **Leigh M. Vanderloo:** Writing – review & editing, Investigation, Funding acquisition, Conceptualization. **Magdalena Janus:** Writing – review & editing, Investigation, Funding acquisition, Conceptualization. **Jonathon L. Maguire:** Writing – review & editing, Supervision, Resources, Investigation, Funding acquisition, Conceptualization. **Catherine S. Birken:** Writing – review & editing, Supervision, Resources, Methodology, Investigation, Funding acquisition, Conceptualization.

## Code availability

Codes are available upon request by contacting www.targetkids.ca/contact-us/. The full code is not freely available to respect the confidentiality of our participants, ensure data integrity, and avoid scientific overlap between projects. Once initial contact has been made, we request a short research proposal which will be subject to review by the TARGet Kids! Scientific Committee and approval by institutional IRBs.

## Funding

This work was supported by 10.13039/501100000024Canadian Institutes of Health Research (CIHR) [grant number #477187].

## Declaration of competing interest

The authors declare that they have no known competing financial interests or personal relationships that could have appeared to influence the work reported in this paper.

## Data Availability

Data will be made available on request.
